# Tissue Specific Profiling of Females of *Schistosoma japonicum* by Integrated Laser Microdissection Microscopy and Microarray Analysis

**DOI:** 10.1371/journal.pntd.0000469

**Published:** 2009-06-30

**Authors:** Geoffrey N. Gobert, Donald P. McManus, Sujeevi Nawaratna, Luke Moertel, Jason Mulvenna, Malcolm K. Jones

**Affiliations:** 1 Queensland Institute of Medical Research, Herston, Queensland, Australia; 2 School of Veterinary Sciences, The University of Queensland, Brisbane, Queensland, Australia; Uniformed Services University, United States of America

## Abstract

**Background:**

The functions of many schistosome gene products remain to be characterized. A major step towards elucidating function of these genes would be in defining their sites of expression. This goal is rendered difficult to achieve by the generally small size of the parasites and the lack of a body cavity, which precludes analysis of transcriptional profiles of the tissues in isolation.

**Methodology/Principal Findings:**

Here, we describe a combined laser microdissection microscopy (LMM) and microarray analysis approach to expedite tissue specific profiling and gene atlasing for tissues of adult female *Schistosoma japonicum*. This approach helps to solve the gene characterization “bottle-neck” brought about by acoelomy and the size of these parasites. Complementary RNA obtained after isolation from gastrodermis (parasite gut mucosa), vitelline glands and ovary by LMM were subjected to microarray analyses, resulting in identification of 147 genes upregulated in the gastrodermis, 4,149 genes in the ovary and 2,553 in the vitellaria.

**Conclusions:**

This work will help to shed light on the molecular pathobiology of this debilitating human parasite and aid in the discovery of new targets for the development of anti-schistosome vaccines and drugs.

## Author Summary

Schistosomes are parasitic worms responsible for important human diseases in tropical and developing nations. There is urgent need to develop new drugs and vaccines to augment current treatments for this disease. In recent years, concerted efforts by many laboratories have led to extensive genetic sequencing of the parasites, and the publication of genome sequence for two agents of schistosomiasis appears imminent. This genetic information has revealed many molecules expressed by the schistosome parasites for which no functional information is available. This lack of information extends to ignorance of where in the complex multicellular schistosome parasites the genes are expressed. We integrated two molecular and cellular techniques to address these knowledge gaps. We used laser microdissection microscopy to dissect small but highly important tissues involved in nutrition and reproduction from sections of female *Schistosoma japonicum*. From these dissected tissues we then used a broad molecular biology method to identify the multiple genes active in these tissues. Our approach has allowed us to formulate the basis of a “gene atlas” for schistosome parasites, defining the expression repertoire of specific tissues. The better understanding of the roles of tissues in parasite biology, especially in development, reproduction and interactions with its human hosts, should promote future investigations into pathogenesis and control of these significant parasites.

## Introduction

Members of the genus *Schistosoma* are parasitic blood flukes responsible for the serious but neglected human disease of schistosomiasis [1],[2]. In common with other platyhelminths, schistosomes exhibit acoelomy, the body plan characteristic of basal bilaterians whereby tissues are bound together by cells and matrices of the parenchyma in the absence of a body cavity. This body organization, together with the generally small size of adults and developing stages, has been a major hindrance for functional analyses of individual schistosome tissues and cells, because it has been impossible to isolate them. These problems are exacerbated by poor knowledge and limited annotations of many schistosome genes and the absence of basic knowledge of where, and when, in development the molecules are expressed. Localization methods incorporating immunocytochemistry and *in situ* hybridization have been at the vanguard of functional studies of schistosome proteins [3], but the prospect of obtaining robust, informative localization data of multiple genes expressed throughout the complex schistosome life cycle remains a daunting challenge.

Concerted international efforts have been directed at defining functional relevance of the predicted 14–16,000 schistosome genes to identify potential targets for drug and vaccine therapies [4],[5]. Release of extensive schistosome ESTs (Expressed Sequence Tags) datasets and the anticipated publication of complete genomes for *Schistosoma mansoni* and *S. japonicum* [5],[6],[7] have provided new stimulus to achieving these goals. These datasets have enabled development of platforms for transcriptome and proteome analyses to explore gender, developmental and strain differences in schistosomes [8],[9],[10],[11],[12],[13],[14],[15],[16],[17],[18].

Here, we report on tissue-specific gene expression analysis of adult female *Schistosoma japonicum*, as a means to expedite functional characterization of schistosome gene products. Our approach incorporates methods of laser microdissection microscopy (LMM) to generate tissue-specific transcriptional extracts for subsequent microarray analysis. This work follows hypotheses [19],[20] that LMM would prove an excellent means to expedite transcriptional typing of many schistosome tissues despite the acoelomate body plan of these parasites.

## Materials and Methods

### Parasite isolation and sample preparation

The use of mice in this study was approved under Project P288 by the Animal Ethics Committee of the Queensland Institute of Medical Research. *Schistosoma japonicum*-infected *Oncomelania hupensis hupensis* snails, collected from Anhui Province, China, were provided by the National Institute of Parasitic Diseases-CDC, Shanghai. Adult worm pairs were perfused 6 weeks post-challenge from infected ARC Swiss mice. Two batches of approximately 25 live female parasites were flat embedded in Tissue-Tek Optimal Cutting Temperature compound (OCT) (ProSciTech, Australia) and snap-frozen on dry ice. The sample blocks were stored at −80°C, prior to sectioning with sterile blades in a cryostat. Sections were cut at 7 µm and mounted onto a sterilized polyethylene-naphthalene membrane on a microscope slide (P.A.L.M. Microlaser Technologies, Germany). The slides were then stored at −80°C.

For transmission electron microscopy, female parasites were fixed in 3% glutaraldehyde in 0.1 M phosphate buffer at pH 7.4 for 2 h, post-fixed in potassium ferricyanide-reduced osmium tetroxide, followed by 5% aqueous uranyl acetate, dehydrated in acetone and embedded in Epon resin. Ultrathin sections were viewed on a JEM 1011transmission electron microscope operated at 80 kV.

### Laser microdissection microscopy (LMM)

Thawed cryo-sections were fixed immediately in 100% methanol for 30 seconds, stained with 1% Toluidine blue (CHROMA, Germany) for 10 seconds, washed in diethylpyrocarbonate (DEPC)-treated water, 2×10 seconds, and allowed to dry for 20–30 min before microdissection. A PALM microbeam laser catapult microscope (P.A.L.M. Microlaser Technologies) was used to microdissect the gastrodermis from posterior regions of female worms, and the ovary and vitelline tissues from the stained frozen sections. An area of approximately 4 million squared µm (approximately 20×10^6^ µm^3^ of tissue) was collected separately from each of the tissues onto 500 µl opaque adhesive caps (P.A.L.M. Microlaser Technologies). The areas amount to the collection of many 1000's of microdissected elements for each tissue. For control tissue, we used 12 *S. japonicum* females that were snap-frozen in OCT and sectioned by cryostat. Control sections were collected onto 6 sterile glass slides. Entire sections were then scraped by sterile scalpel blades from the slides into RNA extraction buffer (below) for analysis. The control samples therefore represent the transcriptional repertoire of entire females.

### Total RNA isolation and hybridisation

Total RNA was isolated from the control and LMM samples using RNAqueous-Micro kits (Ambion) kit using the manufacturer's instructions and quantified using a Nano-Drop ND-1000 spectrophotometer (Thermo Scientific, USA). The quality of total RNA was assessed using a Bioanalyser RNA Pico Lab Chip (Agilent) prior to storage at −80°C.

### Microarray hybridisation and feature extraction

Full details of the design and construction of the schistosome microarray used have been reported [11]. In brief, the array was constructed from information based on the transcriptomes of adult *S. japonicum* and *S. mansoni*. The microarray consists of 19,222 target contiguous sequences (contigs) printed twice from two independent probe designs, and includes 12,166 probes derived from *S. mansoni*, and 7,056 probes derived from *S. japonicum*. An overview of the design and composition of the microarray is present in Table S1.

A 300 ng aliquot of total RNA from each sample was converted into complementary RNA was synthesized and labeled with the fluorophore Cyanine 3-CTP (CY3c) and hybridized according to the manufacturer's instructions (Agilent Technologies -One-Color Microarray-Based Gene Expression Analysis). Microarray hybridisations were performed in duplicate for all samples. The yield, concentration, amplification efficiency and abundance of CY3c were measured at A*260* and A*550* by spectrophotometry.

### Data analysis

Hybridized slides were scanned using an Agilent Microarray Scanner (B version) as tiff files and processed with the Feature Extraction 9.5.3.1 Image Analysis program (Agilent) to produce standardised data for statistical analysis. All microarray slides were assessed for background evenness by viewing the tiff image by Feature Extraction. Feature extracted data was analysed using GENESPRING (version 7.3.1; Agilent Technologies/Silicon Genetics, Redwood City, CA).

Microarray data were normalised using a normalisation scenario for “Agilent FE one-color” which including “Data Transformation: Set measurements less than 5.0 to 5.0”, “Per Chip: Normalize to 50th percentile” and “Per Gene: Normalize to median”. Data sets were further analysed using published procedures based on one-colour experiments [21]. The gProcessedSignal values determined in GENESPRING using Agilent's Feature Extraction software including aspects of signal/noise ratio, spot morphology and homogeneity. Thus, gProcessedSignal represents signal after localised background subtraction and includes corrections for surface trends. Features were deemed *Absent* when the processed signal intensity was less than two fold the value of the processed signal error value. Features were deemed *Marginal* when the measured intensity was at a saturated value or if there was a substantial amount of variation in the signal intensity within the pixels of a particular feature. Features that were not *Absent* or *Marginal* were deemed *Present*. Data points were included only if *Present* or *Present*, *Absent* and probes or contigs retained if all data points were *Present* or *Present*, *Absent*.

Microarray data have been submitted to the Gene Expression Omnibus public database, under accession numbers GPL7160 and GSE12706.

### Gene ontology analysis

Batch BlastX (6 frame translation protein homology) was performed at http://www.blast2go.de on all contigs. This presented a further overview of the gene ontologies that are modulated between tissue types in adult female *S. japonicum* (Figure S1 and Table S2). This information was used to supplement previously published GOs based on nucleotide sequence [11]. To gain a more complete overview of the GO categories that are modulated during the *S. japonicum* lifecycle we used the software ErmineJ to produce extended list of GOs associated with each of the microdissected tissue types [22].

### Real time PCR validation of microarray data

A total of 9 gene sequences indentified as differentially expressed among the three *S. japonicum* tissues and whole worm control tissue were chosen for validation of microarray data using real time PCR as described [12]. The template for real time PCR was that obtained by microdissection. Forward and reverse primers (Sigma-Aldrich, Australia) were designed from the 10 contigs (Table S3). All total RNA samples were DNase treated (Promega, Australia) prior to synthesis of cDNA using a QuantiTect Whole Transcriptome Kit (QIAGEN, Australia). All cDNA samples were diluted to a concentration of 5 ng/µl. Real time PCR was performed in a Gene Disc 100 ring (Corbett Research, Australia). A sequence from the NADH-ubiquinone reductase gene of *S. japonicum* was used for normalisation of data from all experiments. Each experiment was performed in duplicate, and the confidence threshold (CT) of the second set was normalised to the first set before evaluation. This was done by importing the standard curve of the first set to that of the second using Rotor Gene 6 software [12]. Microarray and real time PCR datasets were tested following Morley and colleagues [23]. Data were analysed using Graphpad Prism Version 5. Data from microarray and real time PCR populations were examined to ascertain if they fit normal distributions, using the D'Agostino and Pearson omnibus normality test and the Shapiro-Wilk normality test. Because both sets of data were not normally distributed, a Spearman correlation (Rho) was employed to test for correlation. The statistical analyses used an alpha value of 0.05.

## Results/Discussion

**Figure 1:**
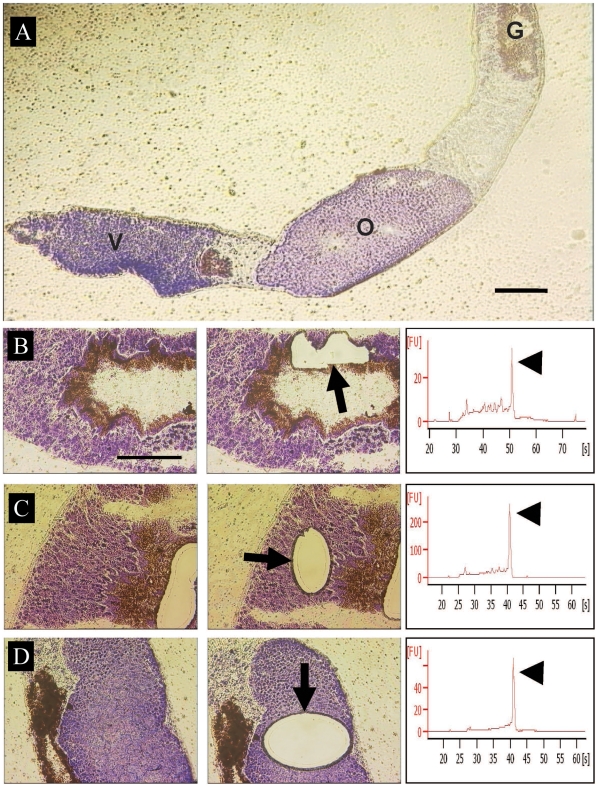
Laser microdissection of *S. japonicum* tissues. (A) Longitudinal section through female, showing morphology of gastrodermis (G), ovary (O), and vitelline tissue (V) from toluidine blue-stained cryostat sections. Bar = 100 µm. (B) Gastrodermis. Bar = 100 µm (C) Vitellarium and (D) Ovary; before (left panels) and after (centre) LMM. For each tissue, the region of microdissected tissue is indicated with an arrow. The panel on the right shows quality of total RNA from the three microdissected tissues determined using a Bioanalyzer. The prominent 18S ribosomal RNA band is indicated by the arrowhead and is an indication of the fidelity of the total RNA. A distinct 28S band is never visible in total RNA fractions of schistosomes.

**Figure 2:**
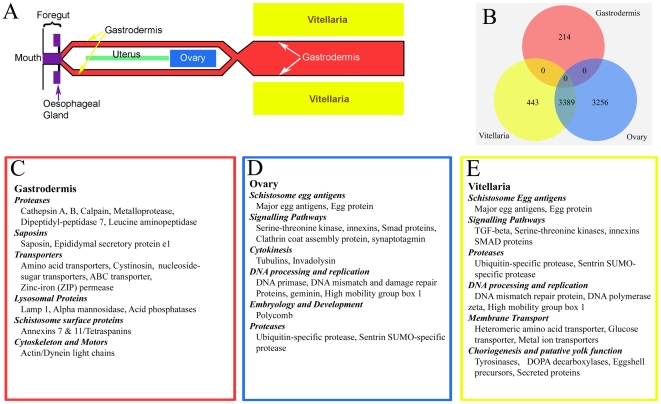
Microarray analysis of microdissected tissues of female *S. japonicum*. (A) A two-dimensional model of the arrangement of major organ systems of female schistosomes, showing relative location of the three tissues dissected here. (B) Venn diagram outlining the number of probes that were detected at 2 fold or greater in each tissue type and the degree of overlap in expression of those genes between and among tissues. (C–E). Lists of example genes enriched for each tissue. These lists are selected from full lists (Table S4).

We targeted three female tissues, namely, gastrodermis (absorptive gut lining) of the posterior halves of the worms, ovary, and the vitelline glands ( = vitellaria, accessory glands of the female system that produce precursors for eggshell synthesis) (Figures 1 and 2). We chose these three tissues due to their relative abundance, clearly delimited structure and the important biological roles in schistosome development and reproductive biology.

**Figure 3:**
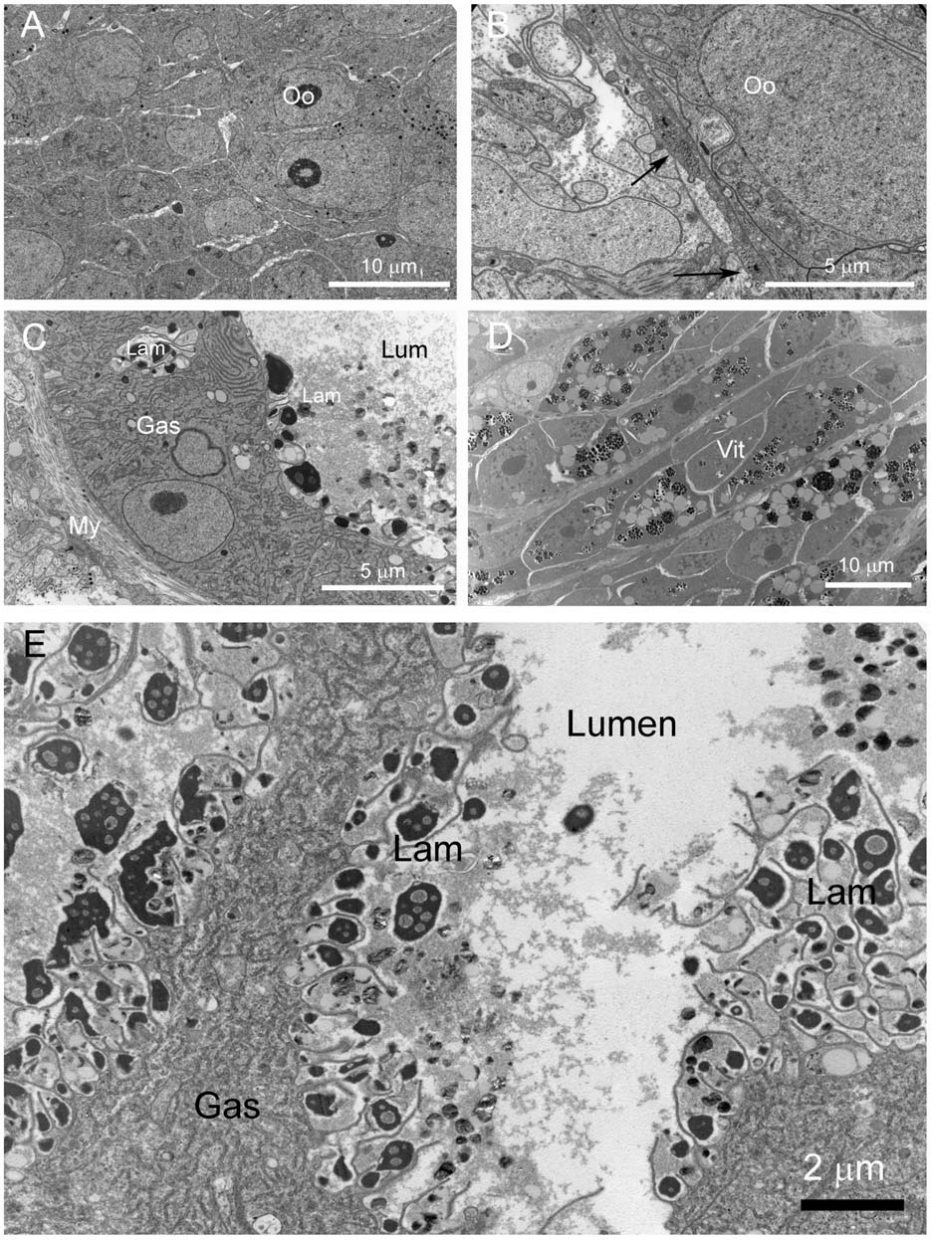
Ultrastructural morphology of microdissected tissues of female *S. japonicum*. (A) Ovary. Numerous oocytes with a high nuclear-cytoplasmic ratio are present as the sole cell type. (B). A thin cellular layer, incorporating myofibrils, is present as the limiting margin of the ovary. Arrow indicates margin of ovary. (C) Gastrodermis. The gastrodermis is a unilaminate syncytial layer forming the absorptive lining of the gut. (D). Vitelline cells. These accessory cells of the female reproductive system secrete egg-shell precursors and possibly yolk. Electron opaque lipid droplets are prominent features of this region. (E). Luminal surface of gastrodermis showing multiple stacked lamellae. Caption abbreviations; Gas = Gastrodermis epithelium, Lam = Lamellae, Lum = Lumen, My = Smooth muscle fibres, Oo = Oocyte, Vit = Vitelline cell.

In view of the closely knit organization of schistosome tissues, it was important to know whether the three tissues under investigation represented homogenous cell populations. Ultrastructural assessment indicated that the ovary and gastrodermis were homogenous (Figure 3). We had previously shown through ultrastructural studies incorporating a stereological analysis of the relative volumes of tissues in vitellogenic regions that although some parenchymal tissues intrude into the vitelline regions, vitellogenic regions are dominated by vitelline cells (vitellocytes) [24] which are highly synthetic cells. Thus, all tissue extracts represent homogenous or near homogenous samples.

For microarray analysis, unfixed frozen females were sectioned by cryostat onto membrane-coated slides, stained with toluidine blue and microdissected using a PALM laser catapult microscope (Figure 1). Total RNA integrity from microdissected samples was assessed (Figure 1) and shown to be of high fidelity. A distinct 28S band is never visible in total RNA fractions of schistosomes [25]. RNA was further processed for one-colour fluorophore-labelled cRNA synthesis and hybridization to a microarray representing the near complete transcriptome of adult schistosomes [11]. Of 38,444 probes (representing 19,222 contigs) on the chip, 8,454 (5,242 contigs) were retained after filtering (Table S4).

Principal component analysis (PCA) is a multi-dimension reduction method that allows the visual presentation of a complex data set, so that distances between plotted points represents the relative similarity of each datasets. Usually plotted in an X,Y,Z formation, each axis represents a distinct subset of data points, or in the current application, gene lists. Gene expression profiles of the three microdissected tissues and the control sample were analysed by PCA (Figure S2). The point of the control tissue was more similar to those of the gastrodermis and vitellaria, compared with the ovary. This observation is not surprising, for the former tissues are voluminous in female parasites and likely account for much of the female transcriptome.

Complete lists of genes enriched for each tissue sample after normalization, together with lists of selected genes of interest enriched for each tissue are presented (Tables 1–3, Table S4, and Figure 2). Major gene ontologies (GOs) of differentially expressed genes for the three tissues are also shown (Figure S1 and Table S2). Abundant transcripts enriched for each tissue encoded protein sequences for which there was little or no annotation or sequence identity.

**Table 1:** Examples of differentially expressed genes of the gastrodermis of *S. japonicum* normalised to signal intensity of the vitelline and ovary tissues.

Systematic Name	Probe	Annotation Microarray	Protein Homology	Fold Change
Contig5007	_1	SJCHGC04509	PV-fam-domain, with meprin domain	230.9
Contig7517	_899	SJCHGC00284	epididymal secretory protein e1	92.8
Contig7606	_687	SJCHGC02336	Cathepsin A	81.7
Contig7602	_446	SJCHGC09134	Lysosome-associated membrane glycoprotein (Lamp)/CD68	46.8
Contig4694	_436	*Schistosoma japonicum* mRNA for calpain	Calpain	33.2
Contig1623	_1100	SJCHGC05100	ABC transporter	31.3
Contig5864	_1109	heme maturase [Tetrahymena pigmentosa]	Heme maturase	17.4
Contig7648	_532	SJCHGC01821	Purple acid phosphatase, N-terminal	13.2
Contig8540	_456	SJCHGC02844	CD9 antigen/tetraspanin	10.7
Contig2584	_847	Caenorhabditis elegans cosmid C23H4	Domain: Cystinosin/ERS1p repeat	10.6
Contig8609	_1232	SJCHGC09591	Prostatic acid phosphatase/histidine acid phosphatase	6.2
Contig6992	_996	SJCHGC05833	Phosphatidic acid phosphatase/chloroperoxidase, N-terminal	5.8
Contig3173	_531	SJCHGC09122	Epidermal growth factor receptor	4.4
Contig4589	_431	SJCHGC04027	Dynein heavy chain domain 3	4.3
Contig5962	_876	*S.japonicum* mRNA for cathepsin B	Cathepsin B	3.6
Contig8263	_2582	*Schistosoma mansoni* myosin heavy chain (MYH)	Myosin heavy chain	3.4
Contig7700	_1006	*Schistosoma japonicum* clone ZZD1392	Tegumental protein 31.8 kDa [Clonorchis sinensis]Dynein light chain, type 1 and 2	3.2
Contig5394	_765	SJCHGC02330	novel transmembrane amino acid transporter protein	2.9
Contig8017	_602	SJCHGC06760	Annexin a7	2.8
Contig6015	_909	SJCHGC01645	Alkaline-phosphatase-like	2.5
Contig6810	_904	SJCHGC05604	Zinc/iron permease	2.5
Contig1093	_646	SJCHGC06386	Permease for cytosine purines uracil thiamine allantoin	2.0

A full list of genes, including systematic name and probe identification, expressed by the gastrodermis is shown in Table S4. Fold change refers to expression relative to ovary and vitellaria.

**Table 2 pntd-0000469-t002:** Examples of differentially expressed genes of the ovary, normalised to signal intensity of the gastrodermis.

Systematic Name	Probe	Annotation Microarray	Protein Homology	Fold Change
Contig5637	_571	SJCHGC03728	Unknown	336.6
Contig6302	_526	SJCHGC04563	Clathrin coat assembly protein ap19	135.2
Contig3450	_554	*Lymnaea stagnalis* synaptotagmin I mRNA	synaptotagmin I	63.5
Contig1394	_190	SJCHGC06831	Innexin	60.1
Contig8876	_2764	SJCHGC06324	Major egg antigen	38.1
Contig1301	_18	5′ end of clone FK0AAA23AE07 (strain 6–9), *Anopheles gambiae*	Geminin isoform cra_a	37.5
TC11333	_1147	Weakly similar to DNA mismatch repair protein MSH2 - African clawed frog	Mismatch repair protein	32.6
Contig6946	_1090	SJCHGC05810	Serine threonine kinase cdc2	22.9
TC8161	_669	Similar to DNA polymerase epsilon catalytic subunit A	DNA polymerase epsilon catalytic subunit	16.8
TC18876	_4354	SJCHGC08812	DNA polymerase epsilon small subunit	16.1
Contig8250	_2443	*Schistosoma japonicum* preprocathepsin L mRNA, complete cds	Preprocathepsin L	13.1
Contig8644	_1259	SJCHGC01849	DNA-damage repair protein drt111 precursor	12.4
TC17330	_737	SJCHGC05965	Proliferating cell nuclear antigen	11.7
Contig2662	_759	SJCHGC05418	Sentrin sumo-specific protease	10.2
Contig8918	_1226	Schistosoma japonicum clone ZZZ431 mRNA sequence	Egg protein cp422	10.1
Contig6677	_676	SJCHGC04972	Polycomb homologue	9.1
Contig5392	_935	SJCHGC02371	Peter pan homolog	8.8
TC10915	_564	Similar to similar to GenBank Accession Number U00997 synaptobrevin in *Aplysia californica*, partial	Synaptobrevin	8.7
Contig7167	_399	*Limulus polyphemus* syntaxin 1C mRNA, complete cds/Hypothetical protein T26C11.2 [*Caenorhabditis elegans*]	Syntaxin 1a cg31136-pa	8.0
Contig6123	_699	*Schistosoma mansoni* Smad4 (Smad4) mRNA, complete cds	Smad4	7.2
Contig6819	_526	*Aquifex aeolicus* VF5 section 109 of 109 of the complete genome	DNA repair endonuclease	6.7
Contig5796	_486	SJCHGC04715	Major facilitator superfamily domain containing 8	6.6
TC7109	_2083	SJCHGC02245	enhancer of polycomb homolog 1	6.2
Contig5374	_1436	*Schistosoma mansoni* Smad1 (Smad1)	Smad1	6.1
TC13948	_859	Receptor tyrosine kinase (*Schistosoma mansoni*), partial (92%)	Tyrosine protein kinase	6.1
Contig5026	_422	SJCHGC06696	DNA primase	4.0
TC9260	_582	Hypothetical protein [*Plasmodium falciparum* 3D7]- PF14_0537	DNA repair helicase	2.3

A full list of genes, including systematic name and probe identification, expressed by the ovary is shown in Table S4. Fold change refers to expression relative to gastrodermis.

**Table 3:** Examples of differentially expressed genes of the vitelline glands, normalised to signal intensity of the gastrodermis.

Systematic Name	Probe	Annotation Microarray	Protein Homology	Fold Change
Contig7083	_1167	SJCHGC03760	protein serine threonine kinase	8.8
Contig5547	_760	Schistosoma japonicum clone ZZD46 mRNA sequence	Protein tyrosine phosphatase domain containing 1 protein	8.5
Contig8039	_772	SJCHGC01089	hypothetical protein containing signal peptide	8.5
Contig6705	_319	Medicago truncatula clone mth2-32e10	DOPA decarboxylase	2.1
Contig5142	_489	SJCHGC04289	Solute carrier family 7 (cationic amino acid system) member 8	6.9
Contig3412	_1137	*Schistosoma japonicum* clone ZZD254 mRNA sequence	Beta–n-acetylglucosaminyl transferase 5	6.8
Contig8876	_2764	SJCHGC06324	Major egg antigen	6.6
Contig 8365	_797	*Schistosoma japonicum* clone ZZZ329 mRNA sequence	tyrosinase	6.2
Contig6381	_463	*Schistosoma japonicum* clone ZZD128 mRNA sequence	Schistosome venom allergen-like protein	6.0
Contig6709	_725	SJCHGC06813	Protein-4.1 G protein	5.1
Contig5541	_663	*Botrytis cinerea* strain T4 cDNA library under conditions of nitrogen deprivation	Sjchgc02267 protein- similar to pannexin	4.6
Contig8457	_1851	SJCHGC01511	Selenoprotein w- eggshell precursor or fs800	4.0
Contig6208	_611	SJCHGC06498	Solute carrier family member 4	3.5
Contig7264	_1186	TGF-beta	Transforming growth factor-beta	3.3
Contig4028	_618	SJCHC06704	Innexin	3.0
Contig3752	_1130	SJCHGC03615	Tyrosine kinase 5	2.8
TC13948	_859	receptor tyrosine kinase (*Schistosoma mansoni*), partial (92%)	Tyrosine protein kinase	2.7
Contig1512	_665	*Schistosoma mansoni* immunophilin FK506 binding protein FKBP12 mRNA, complete cds	Immunophilin	2.0

A full list of genes, including systematic name and probe identification, expressed by the vitelline glands is shown in Table S4.

**Figure 4:**
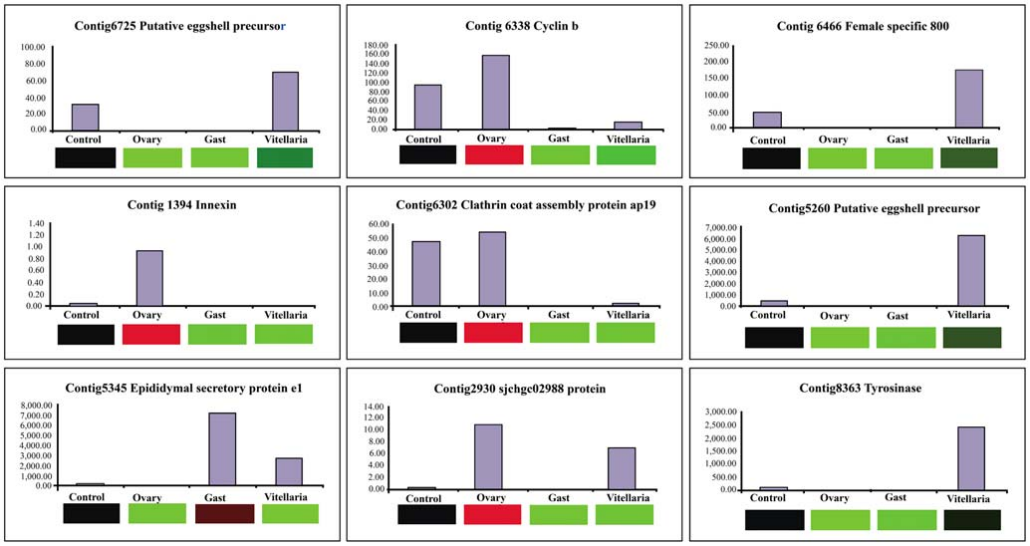
Validation of a subset of differentially expressed genes in the three different microdissected tissues from adult female *S. japonicum* compared with control tissue. The real time PCR data, expressed as copy number, are presented as bar graphs, while the corresponding microarray data are shown below the graphs as heat maps. Microarray gene expression is indicated by up-regulation (Red), down-regulation (Green) or unchanged (Black).

Nine transcripts that were enriched in one of the 3 tissues were selected for validation of expression level by real time PCR using cDNA templates from the microdissected and control samples (Figure 4). Expression levels observed by real time PCR agreed with those by microarray for these genes. The microarray and real time PCR data sets of the 9 genes showed a significant correlation of 0.6791 (Spearman's Rho, p<0.0001, n = 27).

After filtering the microarray data and normalizing signal relative to female germinal tissues, we identified 214 probes representing 147 genes enriched for the gastrodermis (Figure 2, Table 1, and Table S4). Comparable datasets in Table S4 compare gene expression of the gastrodermis relative to either ovary or vitellaria. These three datasets show strong congruence, although with some variation in relative enrichment of some sequences. Thus, a ferritin isoform is enriched in the gastrodermis relative to the ovary, but not relative to the vitellaria (Contig7767, Table S4).

The enriched genes of the gastrodermis relative to female germinal tissues included proteases of the haemoglobinolytic cascade, membrane-associated molecular transporters, actin and associated molecular motors. A highly enriched gene of the gastrodermis, represented by Contig5007, is a hitherto uncharacterized gene with uncertain sequence identity, but which contains motifs with similarity to the meprin family of metalloproteinases, and an erythrocyte-binding protein of malaria parasites. This molecule potentially represents a novel class of proteinases involved in haemoglobinolysis in these vascular parasites [26]. Surprisingly, cathepsin D, an early member of the haemoglobinolysis cascade [27], was not enriched for the gastrodermis. Given its upstream role in this multi-enzyme network, cathepsin D is probably expressed in anterior zones of the gut, either in the oesophageal gland, or in anterior zones of the gastrodermis. Our study focused on microdissection of the posterior regions of the gastrodermis. Regional specialization of the apparently simple gastrodermis of other platyhelminths has been postulated [28]. It may be that the schistosome gut displays a similar planar polarity, evidenced by distinct secretory product in different zones along the length of the parasite [26]. The hypothesis is further substantiated by observations that the gastrodermal regions analysed here were enriched for numerous sequences encoding dipeptidases and carboxypeptidases (Table S4), peptidases more likely to be associated with terminal parts of the haemoglobinolytic cascade.

Additionally, transcripts encoding proteins previously localized to the outer tegumental surface of the parasite (tetraspanins, annexin and alkaline phosphatase) were enriched for the gastrodermis relative to other tissues. Although these molecules have been previously recognised as tegumentary components, their occurrence in the syncytial gastrodermis is not surprising. Transcripts for divalent metal transporters, particularly a member of the Zinc regulated transporter/iron regulated transporter family (ZIP) family were enriched for the gastrodermis. Schistosomes have high dietary requirements for iron [29],[30] and other divalent metals. While a surface mediated pathway for iron uptake by schistosomes has been postulated [29], the presence of metallo-transporters in the parasite gut indicates that this tissue may also scavenge the trace metals [29].

Other transcripts enriched for the gastrodermis (relative to ovary and vitelline tissues) represent genes encoding lysosomal proteins, namely, cystinosin, lysosomal acid membrane glycoprotein (Lamp1/CD68), lysosomal alpha mannosidase and acid phosphatases (Figure 2, Table 1, and Table S4), although lysosomes are not abundant cytoplasmic features of the gastrodermis. A consistent feature of the syncytium, however, is the presence of apical epicellular vacuoles (Figure 3), which enclose parts of ingested host blood, and are lined by villus-like lamellae. The vacuoles have many features of lysosomes, namely a low pH and presence of proteolytic enzymes [26] and are a possible cellular location for the lysosome molecules of the gastrodermis.

We identified 6,645 probes representing 4,149 upregulated genes (Figure 2, Table 2, and Table S4) for the ovary compared with the gastrodermis. Similarly, we identified 3,832 probes representing 2,553 upregulated genes (Figure 2, Table 3, and Table S4) for vitellaria compared with the gastrodermis. Oocytes and vitellocytes, in platyhelminth evolution and ontogeny, are believed to be derived from common progenitor cells [31]. We decided, therefore, to determine whether the tissues have common expression identity that may reflect the common origin of the two tissues. Analysis of expression by Venn diagram indicated substantial overlap in expression between the two germinal tissues, but not with the gastrodermis (Figure 2B). Genes enriched for both cell types included egg-specific genes including major egg antigens and egg protein cp422. The former gene is also abundant in mature eggs of schistosomes [32]. Other genes enriched for the female reproductive tract included those encoding molecules for TGF-β and tyrosine kinase signalling pathways [33],[34], different innexins (gap junction proteins of invertebrates), and a diversity of genes encoding molecules associated with DNA processing, replication, and transcription. With the exception of the egg-specific antigens, the upregulated genes common to the two tissues are involved in cell proliferation and intercellular signalling.

Genes enriched for the ovary (relative to the gastrodermis) included a number encoding proteins associated with cytokinesis, fertilization and coated pit-mediated endocytosis (Table 2). Oocytes express genes with identity to *polycomb*, enhancers of *polycomb*, and *Peter pan* homologues of vertebrates and ecdysozoans [35] (Table 2). *Polycomb* genes, not previously recognized for platyhelminths, repress *Hox* expression in embryogenesis leading to cellular and zonal differentiation in embryos. Discovery of genes involved in embryonic differentiation will provide new insight into developmental cascades in the complex multi-generational schistosome life-cycle, leading in turn to a better understanding of differentiation of the intraovular embryo, the stage primarily responsible for pathogenesis in schistosomiasis.

Expression analysis of vitellaria (Table 3, Figure 2, and Table S4) revealed enriched genes (relative to the gastrodermis) associated with egg-shell synthesis, as well as a range of membrane transporters with affinity for amino acids, metallo-ions and nucleotides. Eggshell precursors, egg-specific proteins and tyrosinases were upregulated as expected for this tissue that provides precursors for choriogenesis [30],[36]. Numerous membrane-spanning transporters and genes encoding proteins for exocytosis were also enriched as were those associated with lipid metabolism. Some transcripts, annotated as containing signal peptides, did not contain abundant tyrosine residues, a prerequisite for eggshell precursors [37], possibly indicating that these molecules function in aspects other than shell formation. Given the essential role of vitellocytes in egg development and embryogenesis, functional characterization of these putative secreted proteins may enhance our understanding of the complexity of egg-shell synthesis and may help resolve long-standing questions about yolk function of vitellocytes [30].

The integration of microarray analysis of LMM-dissected tissues has provided the means to establish a gene expression atlasing strategy for *S. japonicum*, alleviating the technology hurdles imposed by the acoelomate nature of this platyhelminth and expediting localization of multiple genes. Tissue-specific expression profiling has been performed previously for cavitate invertebrates, incorporating LMM or gross dissection methods [38],[39], but this approach demonstrates the feasibility for gene mapping in a platyhelminth, thus serving as an exemplar for similar studies of other basal bilaterians and small organisms. The localization data provided here serves as a novel resource to advance functional studies of many unannotated *S. japonicum* genes, thereby providing a valuable molecular platform to shed light on the complex physiology and biochemistry of schistosomes, the pathogenesis of schistosomiasis, and to develop new treatments and effective interventions for its control.

## Supporting Information

Figure S1 Major Gene Ontologies of genes represented at greater than or equal to a 2 fold in (a) gastrodermis (b) ovary and (c) vitelline tissues. The number of probes in each gene ontology is noted.
(9.80 MB TIF)

Figure S2 Principal Component Analysis of the 7,623 flag filtered genes showing the gene expression profile of control, gastrodermis (gut), ovary and vitellaria of female S. japonicum.
(0.08 MB TIF)

Table S1 Complete lists of contiguous sequences listed in the custom designed schistosome microarray manufactured by Agilent Technologies used in this study. Column A (ProbeID): Unique identifier of probe on the microarray. Column B (Sequence): Nucleotide sequence of the 60mer probe. Column C (EST Sequence): Complete nucleotide sequence of the assembled EST contig. Column D (TargetID): Contig designation for either S. japonicum (Contig) or S. mansoni (TC). Column E (Accessions): Genbank accession number corresponding to the EST sequences. Column F (Description-Nucleotide): BLASTn annotation result based on nucleotide sequence. Column G (GeneSymbols): Designation of primary or secondary probe design to the corresponding contig. Column H (Protein Homology): BLASTX annotation result based on protein sequence. Column G (Gene Ontology): Gene Ontology number and description.
(29.52 MB XLS)

Table S2 Gene Ontology categories from 2 fold or higher differentially expressed gene detected in the three microdissected tissues.
(0.04 MB XLS)

Table S3 Primer Sets for real time PCR validation of a subset of genes that were upregulated in the three microdissected tissues examined.
(0.02 MB XLS)

Table S4 Complete list of differentially expressed genes, shown on separate sheets of an Microsoft Excel File. ALL DATA Sheet: Relative fold change of all contigs normalised to control. Fold change for gastrodermis is normalised to the signals from an average of ovary and vitelline and individually against the two germinal tissues. The fold change for vitellaria or ovary are normalised to the signal from gastrodermis. Legend: Systematic: Probe identifiers. A full list of probes is shown in Table S1. Protein Identifier: BLASTX annotation result based on protein sequence. Normalised data and Flags are shown in separate columns for each tissue type. Fold changes relative to other tissues for the gastrodermis, ovary and vitellaria are shown is successive columns. Synonym: probe identity. Microarray Description: BLASTn annotation result based on nucleotide sequence. Abbreviations: P- Present; A-Absent; g-gastrodermis, o-ovary; v-vitellaria. In other sheets, the lists of enriched probes (Fold change greater than 2; sorted by decreasing fold changes relative to comparative tissues) for each of the three tissue types are presented on separate sheets of this file. Gastrodermis: Transcripts enriched for gastrodermis relative to ovary and vitellaria. In separate columns the fold changes of genes relative to either ovary and vitellaria are shown. Ovary: Transcripts enriched for ovary relative to gastrodermis. Vitellaria: Transcripts enriched for ovary relative to vitellaria. Legend to Sheets 2–6: Systematic: Probe identifiers. A full list of probes is shown in Table S1. Protein Identifier: BLASTX annotation result based on protein sequence. Fold change of transcripts in tissue featured in sheet relative to other tissues. Synonym: probe identity. Microarray Description: BLASTn annotation result based on nucleotide sequence. Abbreviations: g-gastrodermis; o-ovary; v-vitellaria
(17.43 MB XLS)
