# Supplementary figures and images for: Tissue Specific Profiling of Females of Schistosoma japonicum by Integrated Laser Microdissection Microscopy and Microarray Analysis
Source: PLoS Negl Trop Dis. 2009 Jun 30;3(6):e469. doi: 10.1371/journal.pntd.0000469 (PMC2696939; doi:10.1371/journal.pntd.0000469)

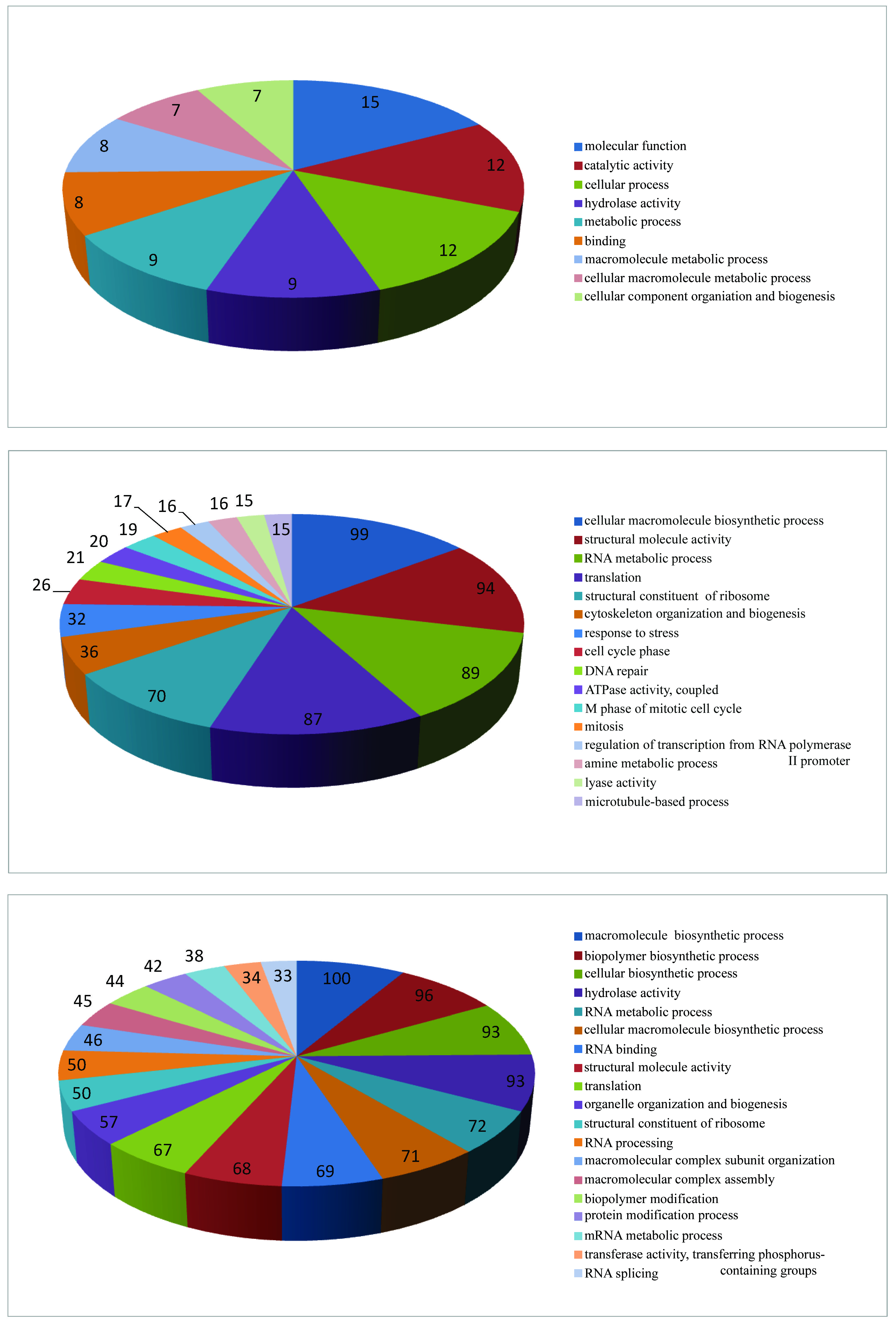

Supplement: Figure S1 — Major Gene Ontologies of genes represented at greater than or equal to a 2 fold in (a) gastrodermis (b) ovary and (c) vitelline tissues. The number of probes in each gene ontology is noted. (9.80 MB TIF) [file pntd.0000469.s001.tif]

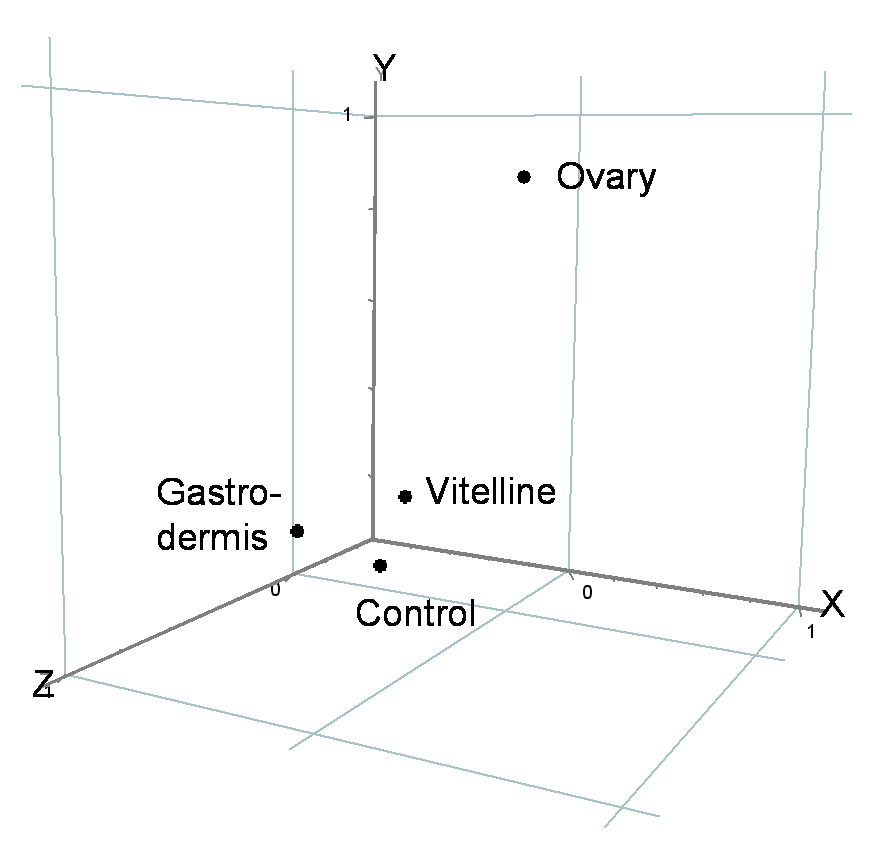

Supplement: Figure S2 — Principal Component Analysis of the 7,623 flag filtered genes showing the gene expression profile of control, gastrodermis (gut), ovary and vitellaria of female S. japonicum. (0.08 MB TIF) [file pntd.0000469.s002.tif]
